# Nutritional and inflammatory biomarkers predicting hospitalization length in acute decompensated heart failure EF below 50%

**DOI:** 10.3389/fcvm.2026.1709763

**Published:** 2026-02-17

**Authors:** Mehmet Özyaşar, Tolga Memioğlu

**Affiliations:** 1 Cardiology Department, Konya City Hospital, Konya, Türkiye; 2 Department of Cardiology, Faculty of Medicine, Izzet Baysal Training and Research Hospital, Bolu Abant Izzet Baysal University, Bolu, Türkiye

**Keywords:** heart failure, HALP score, length of hospital stay, prognostic nutritional index (PNI), systemic immune-inflammation index (SII), systemic inflammation response index (SIRI)

## Abstract

**Background:**

Despite evidence on nutritional and inflammatory markers in various conditions, their combined impact on outcomes in acute decompensated heart failure (ADHF) with reduced left ventricular ejection fraction (LVEF <50%) remains underexplored. This study investigated associations between these biomarkers and clinical outcomes in ADHF patients with LVEF <50%.

**Methods:**

This retrospective study included 232 patients with ADHF hospitalized between January and December 2024. Patients were grouped by hospitalization length (0–5 vs. > 5 days). Nutritional and inflammatory biomarkers were calculated from admission blood samples and analyzed with demographic and clinical characteristics using appropriate statistical methods.

**Results:**

Patients with prolonged hospitalization (>5 days, *n* = 109) had higher Urea, Creatinine, CRP, TroponinT, WBC, Neutrophils, SII, and SIRI, and lower Albumin, Sodium, Hemoglobin, Lymphocytes, HALP, and PNI compared to shorter stays (0–5 days, *n* = 123; *p* < 0.05). All in-hospital deaths occurred in the prolonged group (*n* = 15; *p* < 0.001). Multivariate logistic regression identified higher HALP (OR = 0.987, 95% CI = 0.974–0.999, *p* = 0.033) and PNI (OR = 0.715, 95% CI = 0.644–0.793, *p* < 0.001) as independent predictors of shorter hospitalization. ROC analyses demonstrated PNI (cut-off = 40.51, AUC = 0.85, sensitivity 77%, specificity 77%) outperformed HALP (cut-off = 0.3585, AUC = 0.679, sensitivity 63%, specificity 63%) in predicting shorter stays. SII (cut-off = 983.60, AUC = 0.622) and SIRI (cut-off = 2.917, AUC = 0.626) were associated with prolonged hospitalization but showed limited predictive accuracy.

**Conclusion:**

In ADHF patients with LVEF <50%, poor nutritional status (low HALP and PNI) and high inflammatory burden (elevated SII and SIRI) were linked to prolonged hospitalization. Higher HALP and PNI predicted shorter stays, with PNI showing superior discriminatory power, suggesting nutritional optimization may improve outcomes.

## Introduction

Heart failure (HF) is a complex clinical syndrome characterized by the heart's inability to pump blood effectively, leading to morbidity, mortality, and treatment cost concerns worldwide. In patients with left ventricular ejection fraction (LVEF) below 50%, decompensated HF often necessitates hospitalization, where prolonged hospital stays and increased mortality risk are critical concerns (1). The interplay of nutritional status and systemic inflammation has emerged as a pivotal factor influencing clinical outcomes in HF patients (2). Malnutrition and inflammatory processes exacerbate disease progression, impair immune function, and reduce response to therapy, impacting length of stay in hospital (LOS) and survival (3, 4). In 2016, HF, a clinical syndrome characterized by typical signs and symptoms induced by a structural and/or functional cardiac abnormalities leading to reduced cardiac output was categorized into three subgroups based on LVEF: HF with reduced EF (HFrEF; EF < 40%), HF with mid-range EF (HFmrEF; EF 40%–49%) and preserved EF (EF ≥ 50%) (5).

Nutritional assessment tools, such as the Hemoglobin-Albumin-Lymphocyte-Platelet (HALP) score and the Prognostic Nutritional Index (PNI), provide valuable insights into patients' nutritional status. The Halp score, which integrates parameters like appetite and weight loss, is a practical tool for identifying malnutrition in hospitalized patients (6). Similarly, the PNI, calculated using serum albumin and lymphocyte counts, is a well-validated marker of nutritional and immune status, with lower scores indicating poorer prognosis in cardiovascular diseases (7). Both indices have been associated with adverse outcomes in HF, including prolonged hospitalization and increased mortality (8).

In parallel, systemic inflammation plays a critical role in HF pathophysiology. Inflammatory indices, such as the Systemic Immune-Inflammation Index (SII) and the Systemic Inflammation Response Index (SIRI), reflect the balance between pro-inflammatory and anti-inflammatory processes. The SII, derived from neutrophil, lymphocyte, and platelet counts, has been linked to worse outcomes in cardiovascular diseases, including HF (9). The SIRI, which incorporates monocyte counts, further refines the assessment of inflammatory burden and has shown prognostic value in various chronic conditions (10). Elevated SII and SIRI scores are associated with heightened inflammatory states, which may contribute to prolonged LOS and higher mortality in decompensated HF (11).

Despite growing evidence on the individual roles of nutritional and inflammatory markers, their combined impact on clinical outcomes in acute decompensated heart failure (ADHF) patients with HFrEF, HFmrEF, and HFpEF remains underexplored. This retrospective study sought to investigate the association between prognostic indicators, such as the hospitalization length, mortality, nutritional and inflammatory biomarkers (Halp, PNI, SII, and SIRI) in ADHF patients with LVEF less than 50%. Understanding these relationships could guide targeted interventions to optimize nutritional support and modulate inflammation, potentially improving patient outcomes.

## Methods

### Study design

This retrospective study was conducted at Konya City Hospital, Turkey, following approval from the Konya City Hospital Ethics Committee and the Scientific Research Review Board (Approval date: 07.11.2024, Reference Number: 16-47, 108/2025). The requirement for informed consent was waived by the Ethics Committee due to the retrospective nature of the study. The study was conducted in accordance with the ethical principles of the Declaration of Helsinki and its amendments. Informed consent was waived due to the retrospective nature of the study. This study was designed as a retrospective study, as the inflammatory and nutritional scores we specified would allow us to obtain preliminary findings regarding the duration of hospitalization in patients with EF below 50% in a reasonable patient population and time period. In this study, 880 patients' medical records were analysed retrospectively, those admitted to the Cardiology Clinic with a diagnosis of ADHF between January 1, 2024, and December 31, 2024. Patients were included in the study if they had a confirmed diagnosis of ADHF based on clinical evaluation, imaging, and laboratory findings consistent with the ESC 2021 guidelines. ADHF patients were identified using the International Classification of Diseases (ICD-10) code I50, filtering those available in the hospital data management system (*n* = 880). In the study, the focus was on the demographic and clinical profiles of patients with LVEF <50%, for whom recurrent and prolonged hospitalizations pose a significant problem, as well as the relationships of nutritional and inflammatory biomarkers with prognosis and mortality. Consequently, clinical conditions, including specific confounding factors detailed below, were excluded from the study.

#### Exclusion criteria

- LVEF ≥50%,
- Patients who underwent coronary angiography due to suspected acute coronary syndrome,
- Those with moderate to severe chronic kidney failure, as known or indicated by biochemical laboratory results (creatinine >2 mg/dL),
- Patients with a known hematological or inflammatory disorder.

After applying these exclusion criteria, 232 patients were included in the final analysis.

In the study, the average LOS for all groups in the dataset was recorded as 6.22 ± 4.16 days, with a range from 2 to 35 days. In the literature, both short (< 3–4 days, Sud et al., 2017 (12) and long (> 7–10 days, Omar et al., 2018 (13) hospital stays have been defined for HF, while the average LOS (4–7 days, Whellan et al., 2011 (14) is also reported. Each study may show unique characteristics compared to the others in terms of the average LOS. Considering the average LOS being 6.22 ± 4.16 days for all cohort, and the characteristics of the dataset distribution, this study established a 6-day cut-off point to enhance statistical robustness. Consequently, patients were classified into two groups based on the average LOS: those hospitalized for 0–5 days (< 6 days, shorter hospitalized group, *n* = 123) and those hospitalized for more than 5 days (≥ 6 days, prolonged hospitalized group, *n* = 109). The demographic, clinical, and laboratory data of both groups were recorded for statistical comparison.

### Biochemical tests, nutritional and inflammatory markers

Biochemical and laboratory data were obtained from the analysis of blood samples collected within the first 24 h of admission. All laboratory parameters were measured using standard units and converted if necessary.

Complete blood counts were employed to derive inflammatory indices, including SII and SIRI. These indices were calculated using the following formulas (15):

- SII: Neutrophils (Neu ×10^3/µL) × Platelets (Plt ×10^3/µL)/lymphocytes (Lym ×10^3/µL)
- SIRI: Neutrophils (Neu ×10^3/µL) × Monocytes (Mono ×10^3/µL)/lymphocytes (Lym ×10^3/µL)
- Nutritional biomarkers were calculated using the following formulas (16):
- Prognostic Nutritional Index (PNI) score: [Albumin (g/dL) × 10 + Lymphocyte (x10^3/µL) × 0.005].
- Hemoglobin, Albumin, Lymphocyte, and Platelet (HALP) score: [Hemoglobin (g/dL) × Albumin (g/dL) × Lymphocyte (x10^3/µL)/ Platelet (x10^3/µL)].

### Transthoracic echocardiography

Echocardiographic data were retrieved retrospectively from reports archived in the hospital's database (Vivid S6 Echocardiography; GE Healthcare). In the cardiology department of our hospital, EF measurements were performed by an expert cardiac imaging team using M-mode and “Modified Simpson” methods (17).

### Statistical analyses

Continuous variables are presented as the mean ± standard deviation, along with the range from minimum to maximum values. Categorical variables are presented as counts and percentages. The Kolmogorov–Smirnov test was utilized to evaluate the normality of the data distribution. For the analysis of categorical variables, Pearson's chi-squared test or Fisher's exact test was applied, depending on the sample size and expected frequencies. For continuous variables, the independent samples t-test was used when the data followed a normal distribution, while the Mann–Whitney U test was applied for non-normally distributed data.

Univariate and multivariate logistic regression analyses were conducted to identify factors associated with clinical outcomes. Inflammatory and nutritional biomarkers, such as SII, SIRI, Halp, and PNI scores, were analyzed alongside demographic and clinical variables, including Age, Gender, Rhythm, Glucose, BUN, ALT, CRP, Troponin, and Echo_EF. Each score was modeled individually in the multivariate logistic regression analyses. Odds ratios (ORs), 95% confidence intervals (CIs), and *p*-values were calculated, and the results were compiled into a single table. Furthermore, additional analyses, such as the area under the curve (AUC) and receiver operating characteristic (ROC) assessments, are presented in separate tables.

Statistical analyses were performed using IBM SPSS Statistics, version 27.0 (IBM Corp., Armonk, NY, USA), with a significance level set at *p* < 0.05. All statistical evaluations were conducted by a trained statistician to ensure accuracy and reliability of the results.

## Results

### Demographic and clinical characteristics

The study included 232 patients with ADHF, comprising (*n* = 122, 52.6%) with HFmrEF and (*n* = 110, 47.4%) with HFrEF. The cohort had a mean age of 69.48 ± 12.27 years (range: 30–94 years), with 138 (59.5%) male and 94 (40.5%) female patients. Patients were categorized into two groups based on LOS: shorter LOS (0–5 days, *n* = 123) and prolonged LOS (≥6 days, *n* = 109). No significant differences were observed between groups in age (67.59 ± 11.56 vs. 71.61 ± 12.75 years, *p* = 0.06), hypertension (44.7% vs. 35.8%, *p* = 0.166), diabetes mellitus (36.6% vs. 39.4%, *p* = 0.654), rhythm (sinus rhythm: 59.3% vs. 59.6%; atrial fibrillation: 40.7% vs. 40.4%, *p* = 0.965), or Echo_EF (39.8 ± 8.23 vs. 37.68 ± 9.31, *p* = 0.111) (Table 1). Notably, a significant gender disparity was observed, with a higher proportion of males in the short LOS group (65.9% vs. 52.3%, *p* = 0.036), suggesting potential sex-specific differences in disease severity or treatment response that warrant further investigation. All *n* = 15 mortalities (6.5% of the cohort) occurred in the prolonged LOS group (*p* < 0.001), highlighting a strong association between extended hospitalization and worse prognosis (Table 1).

**Table 1:** Comparison of demographic and clinical characteristics between hospitalized HF patients.

Variables	Overall (*n* = 232)	Length of hospitalization (days)		*p*-value
		0–5 days (*n* = 123)	>5 days (*n* = 109)	
Age	69.48 ± 12.27 [30–94]	67.59 ± 11.56 [42–93]	71.61 ± 12.75 [30–94]	0.06
Gender				**0** **.** **036** *
Female	94 (40.5)	42 (34.1)	52 (47.7)	
Male	138 (59.5)	81 (65.9)	57 (52.3)	
HT	94 (40.5)	55 (44.7)	39 (35.8)	0.166
DM	88 (37.9)	45 (36.6)	43 (39.4)	0.654
Rhythm				0.965
Sinus	138 (59.5)	73 (59.3)	65 (59.6)	
AF	94 (40.5)	50 (40.7)	44 (40.4)	
Clinical status				**<0** **.** **001** *
Discharge	217 (93.5)	123 (100)	94 (86.2)	
Mortality	15 (6.5)	0 (0.0)	15 (13.8)	
Echo_EF	38.8 ± 8.8 [15–49]	39.8 ± 8.23 [20–49]	37.68 ± 9.31 [15–49]	0.111
HFmrEF	122 (52,6)	70 (56.9)	52 (47.7)	0.161
HFrEF	110 (47,4)	53 (43.1)	57 (52.3)	

Bold values indicate statistically significant results (*p* < 0.05).

* Significant at *p* < 0.05 level, Pearson Chi-Square or Fisher's Exact test.

### Biochemical and biomarker differences

Analysis of biochemical parameters revealed significant differences between the shorter and prolonged LOS groups (Table 2). Patients with prolonged LOS had higher levels of Urea (32.71 ± 17.3 vs. 23.15 ± 10.4 mg/dL, *p* < 0.001), Creatinine (1.37 ± 0.5 vs. 1.16 ± 0.4 mg/dL, *p* < 0.001), C-reactive protein (CRP; 32.21 ± 37.2 vs. 15.42 ± 18.6 mg/L, *p* < 0.001), High-sensitive Troponin T (HsTroponinT; 118.22 ± 151.8 vs. 69.06 ± 87.8 ng/L, *p* < 0.001), White Blood Cell Count (WBC; 10.82 ± 4.1 vs. 9.63 ± 3.4 ×10^3/µL, *p* = 0.032), Neutrophil count (8.21 ± 3.7 vs. 6.81 ± 3.1 ×10^3/µL, *p* = 0.003), Systemic Immune-Inflammation Index (SII; 1710.44 ± 1,709.6 vs. 1,197.89 ± 1,167.9, *p* = 0.001), and Systemic Inflammation Response Index (SIRI; 5.38 ± 4.9 vs. 3.79 ± 4.8, *p* < 0.001). Conversely, they had lower levels of Albumin (3.67 ± 0.46 vs. 4.25 ± 0.3 g/dL, *p* < 0.001), Sodium (136.9 ± 4.2 vs. 138.66 ± 3.3 mmol/L, *p* = 0.001), Hemoglobin (11.75 ± 2.4 vs. 13.41 ± 2.1 g/dL, *p* < 0.001), Lymphocyte count (1.7 ± 1.0 vs. 1.88 ± 0.9 ×10^3/µL, *p* = 0.033), Halp score (0.33 ± 0.25 vs. 0.48 ± 0.29, *p* < 0.001), and PNI scores (36.74 ± 4.6 vs. 42.53 ± 3.7, *p* < 0.001). These findings suggest that increased inflammation and poorer nutritional status are associated with prolonged hospitalization. In other words, nutritional scores reveal a negative correlation with prolonged LOS.

**Table 2:** Comparison of biochemical and hematological parameters between hospitalized HF patients.

Variables	Overall (*n* = 232)	Length of hospitalization (days)				*p*-value
		0–5 days (*n* = 123)		>5 days (*n* = 109)		
		Mean ± Sd.	Min-Max	Mean ± Sd.	Min-Max	
Glucose mg/dL	169.06 ± 90.8	160.02 ± 81.0	71–440	179.26 ± 100.2	51–458	0.216
Urea mg/dL	27.64 ± 14.8	23.15 ± 10.4	7–84	32.71 ± 17.3	7–89	**<0** **.** **001** **
Cre mg/dL	1.26 ± 0.5	1.16 ± 0.4	0.48–4.6	1.37 ± 0.5	0.5–3.5	**<0** **.** **001** **
AST U/L	49.82 ± 78.8	37.58 ± 35.9	9–239	63.65 ± 107.0	7–682	0.132
ALT U/L	40.34 ± 85.3	31.25 ± 33.6	5–239	50.6 ± 118.8	5–956	0.934
Alb g/dL	3.98 ± 0.5	4.25 ± 0.3	2.3–5.2	3.67 ± 0.46	2.1–4.8	**<0** **.** **001** **
Na mmol/L	137.83 ± 3.8	138.66 ± 3.3	131–145	136.91 ± 4.2	125–145	**0** **.** **001** **
K mmol/L	4.66 ± 0.6	4.66 ± 0.5	3.6–6.5	4.67 ± 0.7	3–6.7	0.93
CRP mg/L	23.3 ± 30.0	15.42 ± 18.6	0.6–106.6	32.21 ± 37.2	0.6–92.5	**<0** **.** **001** **
Trop. T ng/L	92.15 ± 124.3	69.06 ± 87.8	6.6–419	118.22 ± 151.8	8.32–976.6	**<0** **.** **001** **
WBCx10^3/µL	10.19 ± 3.7	9.63 ± 3.4	2.45–21.8	10.82 ± 4.1	3.27–23	**0** **.** **032** **
H. globin g/dL	12.62 ± 2.4	13.41 ± 2.1	8.9–19.5	11.75 ± 2.4	7.2–17.9	**<0** **.** **001** *
Neu ×10^3/µL	7.46 ± 3.5	6.81 ± 3.1	1.1–19.51	8.21 ± 3.7	1.97–20	**0** **.** **003** **
Lym ×10^3/µL	1.79 ± 0.9	1.88 ± 0.9	0.4–7.0	1.7 ± 1.0	0.27–5.1	**0** **.** **033** **
Plt ×10^3/µL	246.08 ± 81.0	247.6 ± 80.1	85–623	244.3 ± 82.4	95–496	0.712
Monox10^3/µL	0.8 ± 0.3	0.8 ± 0.3	0.05–2.46	0.81 ± 0.3	0.11–2.3	0.976
SII	1,438.69 ± 1,467.2	1,197.89 ± 1,167.9	97.49–7,487.6	1,710.44 ± 1,709.6	189.2–10,202.1	**0** **.** **001** **
SIRI	4.53 ± 4.9	3.79 ± 4.8	0.25–41.9	5.38 ± 4.9	0.23–28	**<0** **.** **001** **
Halp Score	0.41 ± 0.28	0.48 ± 0.29	0.08–1.6	0.33 ± 0.25	0.03–1.45	**<0** **.** **001** **
PNI Score	39.81 ± 5.0	42.53 ± 3.7	23.01–52.0	36.74 ± 4.6	21.01–48	**<0** **.** **001** **

Bold values indicate statistically significant results (*p* < 0.05).

* Significant at *p* < 0.05 level, Independent Samples T-Test.

** Significant at *p* < 0.05 level, Mann-Withney-U Test.

### Predictive analyses

Univariate logistic regression analysis revealed significant associations between prolonged LOS and higher SII (OR: 1.00, 95% CI: 1.00–1.00, *p* = 0.012), SIRI (OR: 1.077, 95% CI: 1.01–1.14, *p* = 0.021), lower HALP score (OR: 0.978, 95% CI: 0.96–0.99, *p* < 0.001), and lower PNI score (OR: 0.709, 95% CI: 0.64–0.77, *p* < 0.001) (Table 3). In multivariate analysis, adjusted for Age, Gender, Rhythm, Glucose, Urea, ALT, C-Reactive Protein, High-Sensitive TroponinT, and Echo_EF, only HALP (OR: 0.987, 95% CI: 0.974–0.999, *p* = 0.033) and PNI (OR: 0.715, 95% CI: 0.644–0.793, *p* < 0.001) scores remained independent predictors of shorter LOS, underscoring their prognostic value (Table 3). Higher SII and SIRI scores were positively correlated with prolonged LOS but lost significance in multivariate models, suggesting a less predictive role compared to nutritional biomarkers.

**Table 3:** Binary logistic regression analysis results for inflammatory & nutritional scores.

Variables	Logistic regression (univariate)			Logistic regression (multivariate)*		
	Odds ratio	[95%CI]	*P* value	Odds ratio	[95%CI]	*p*-value
SII	1.00	1.00–1.00	**0** **.** **012**	1.00	1.00–1.00	0.307
SIRI	1.077	1.01–1.14	**0** **.** **021**	1.027	0.966–1.091	0.399
Halp Score	0.978	0.96–0.99	**<0** **.** **001**	0.987	0.974–0.999	**0** **.** **033** *
PNI Score	0.709	0.64–0.77	**<0** **.** **001**	0.715	0.644–0.793	**<0** **.** **001** *

OR, odds ratio; CI, confidence interval.

Bold values indicate statistically significant results (*p* < 0.05).

* Significant at *p* < 0.05 level; Adjusted for Age, Gender, Rhythm, Glucose, BUN, ALT, CRP, Troponin, Echo_EF variables.

**Figure 1:**
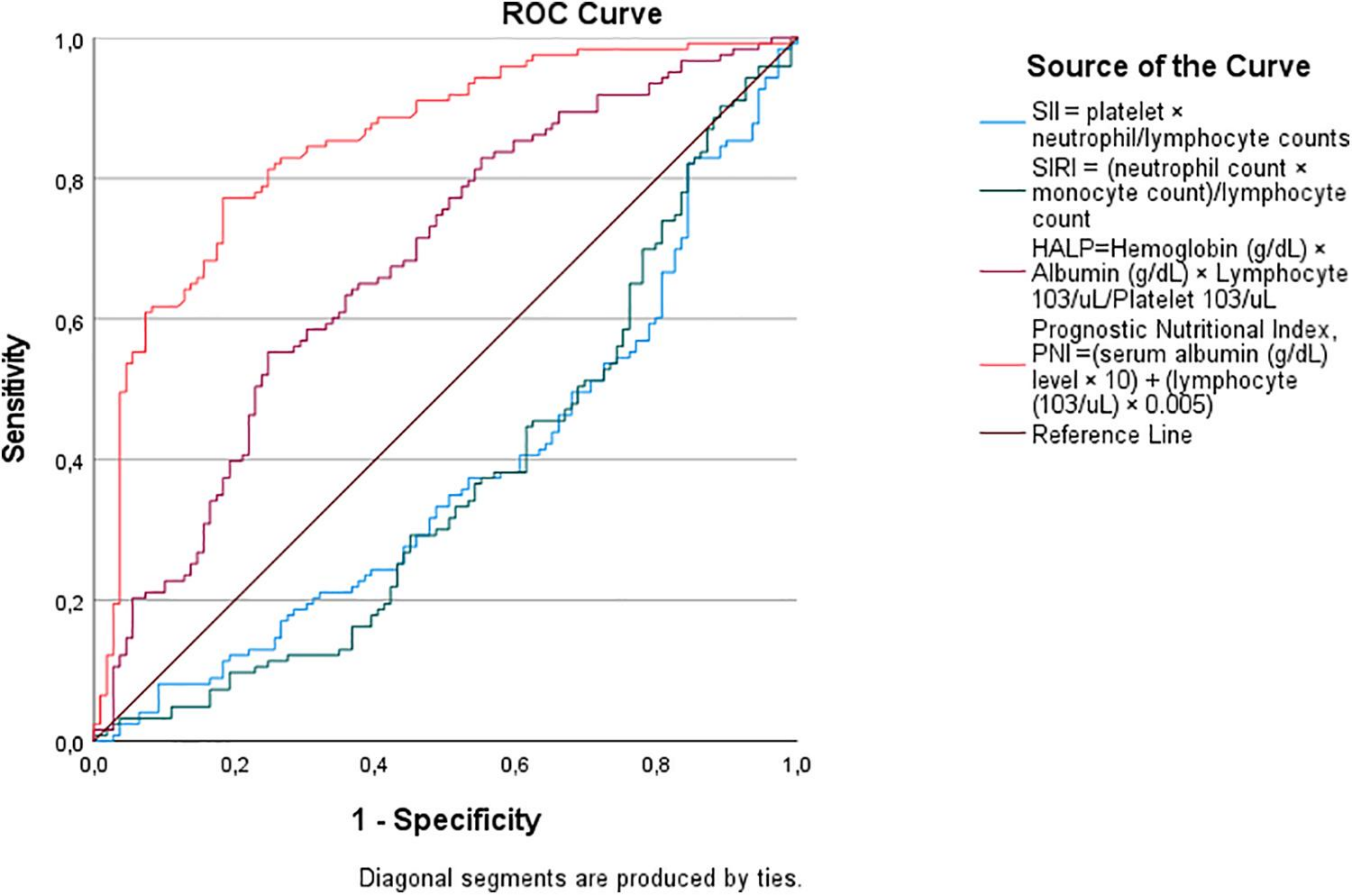
Receiver operating characteristic (ROC) curve analysis for predicting shorter hospitalization (0–5 days). The positive actual state represents patients with 0–5 days length of stay.

**Figure 2:**
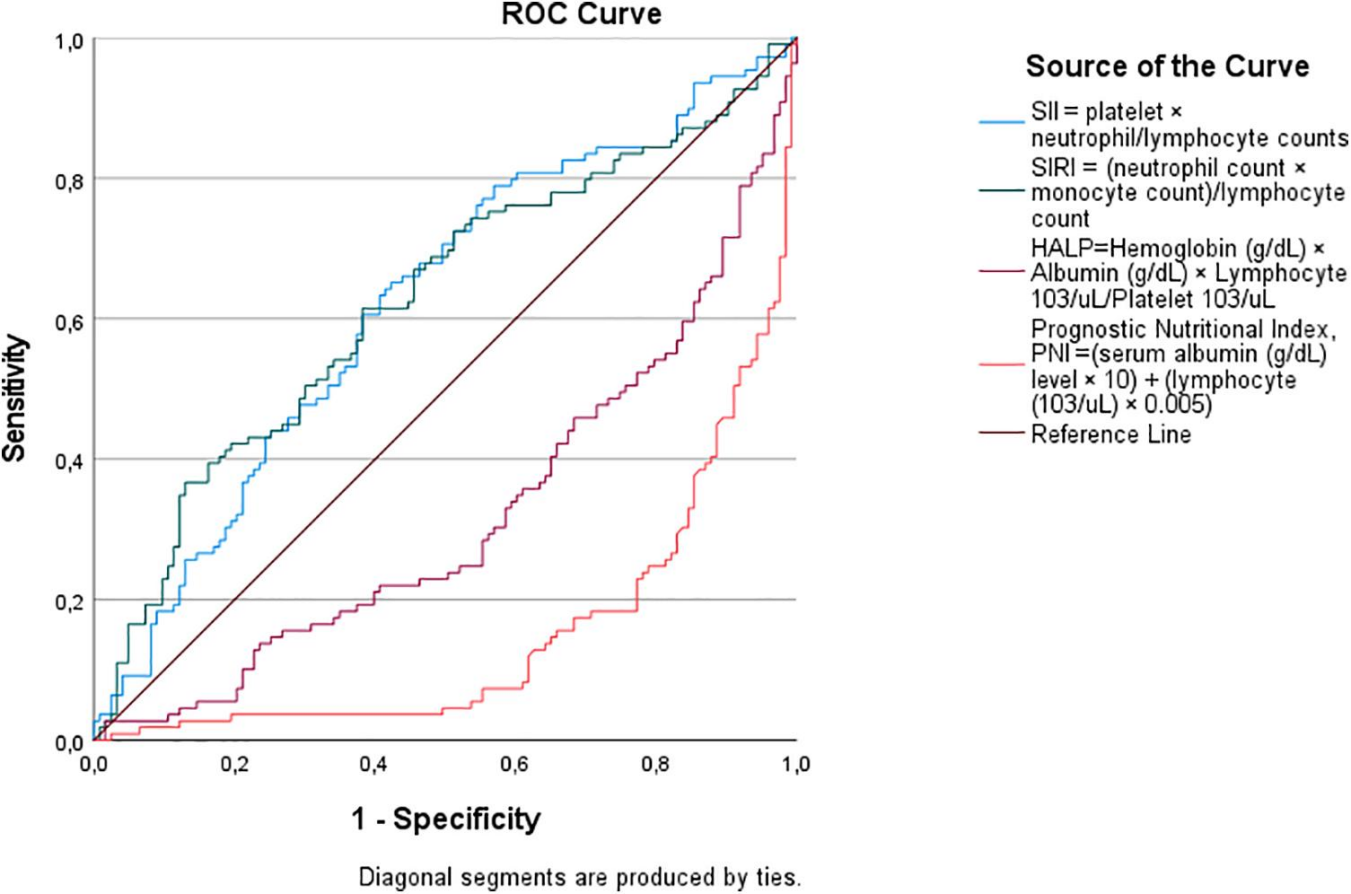
Receiver operating characteristic (ROC) curve analysis for predicting prolonged hospitalization (>5 days). The positive actual state represents patients with >5 days length of stay.

Receiver operating characteristic (ROC) curve analysis confirmed the superior predictive ability of nutritional biomarkers for shorter LOS (Table 4; Figure 1). Higher PNI scores demonstrated greater discriminatory performance, with a cut-off value of 40.51, sensitivity of 77%, specificity of 77%, and an area under the curve (AUC) of 0.850 (*p* < 0.001). Higher HALP scores also showed good predictive performance, with a cut-off of 0.3585, sensitivity of 63%, specificity of 63%, and an AUC of 0.679 (*p* < 0.001). In contrast, SII (cut-off: 983.60, AUC = 0.622, *p* = 0.01) and SIRI (cut-off: 2.917, AUC = 0.626, *p* = 0.01) exhibited modest discriminatory ability for prolonged LOS, as evidenced by their lower AUC values and flatter ROC curves in Figure 2. The steep ROC curve for PNI in Figure 1 visually highlights its superior ability to distinguish shorter from prolonged LOS compared to HALP, SII, and SIRI.

**Table 4:** ROC curve and AUC analysis results of inflammatory & nutritional scores.

Inflammatory scores	Cut-off value	Sensitivity (%)	Specificity (%)	AUC**	Standard error	*P* value*
Halp Score	0.3585	63	63	**0.679**/**(0.321)**	0.035	**<0** **.** **001**
PNI Score	40.5088	77	77	**0.850**/**(0.150)**	0.026	**<0** **.** **001**
SII	983.5965	39	39	**0.378**/**(0.622)**	0.037	**0** **.** **01**
SIRI	2.917	38	38	**0.374**/**(0.626)**	0.037	**0** **.** **01**

Bold values indicate statistically significant results (*p* < 0.05).

* Significant at 0.05 level; Roc Curve and AUC Analysis.

** Calculated as actual state is 0–5 days and >5 days separately.

## Dıscussıon

Length of hospital stay represents a clinically meaningful outcome in ADHF, reflecting not only disease severity and biological vulnerability but also healthcare resource utilization and early prognosis. Prolonged hospitalization has consistently been associated with increased mortality, rehospitalization risk, and healthcare costs (13). In this context, the identification of simple and readily available biomarkers capable of predicting LOS at admission is of substantial clinical value. Our findings demonstrate that nutritional indices, particularly PNI and HALP, can be easily calculated from routine laboratory parameters and applied as practical bedside tools for early risk stratification in ADHF patients with LVEF <50%.

Importantly, the prognostic relevance of these nutritional markers was supported by consistent results across both multivariable logistic regression and ROC analyses. HALP and PNI remained independently associated with shorter hospitalization after adjustment for major clinical and biochemical confounders, while ROC analyses further confirmed their discriminatory capacity, with PNI demonstrating superior performance. This concordance between regression-based and discrimination-based analyses strengthens the robustness of our findings and underscores the potential clinical utility of nutritional assessment in guiding early management strategies in ADHF.

This retrospective cohort study demonstrates that in patients with ADHF and LVEF <50%, poor nutritional status (low HALP and PNI scores) and heightened systemic inflammation (high SII and SIRI scores) are significantly associated with prolonged LOS. Importantly, HALP and PNI independently predicted shorter LOS, with PNI showing superior discriminatory power. These findings highlight the prognostic relevance of nutritional and inflammatory biomarkers in ADHF and align with previous studies emphasizing the interplay of malnutrition and inflammation in HF progression (2, 3, 7, 18, 19).

The association of low HALP and PNI scores with prolonged LOS underscores the impact of malnutrition on HF outcomes. Malnutrition, reflected by reduced albumin, hemoglobin, and lymphocytes, may worsen HF severity by impairing immunity, cardiac repair, and therapeutic response (3, 4). The PNI, integrating albumin and lymphocyte counts, is a validated marker of immune-nutritional status (7). Sun et al. showed lower PNI predicted adverse outcomes in HF (AUC = 0.762), slightly lower than our finding (AUC = 0.850), possibly reflecting the acute nutritional deficits in ADHF with reduced LVEF (7). The HALP score, incorporating hemoglobin, albumin, lymphocytes, and platelets, also reflects nutritional status (6, 16). Koyuncu et al. demonstrated HALP's predictive value for 1-month mortality post-CABG (16). Our results extend this by showing HALP predicted LOS, though with moderate accuracy compared to PNI (Table 4). The stronger performance of PNI may be due to its direct reflection of immune-nutritional balance in acute decompensation (19).

The stronger predictive performance of nutritional indices compared with inflammatory markers may be explained by their ability to capture the cumulative burden of chronic disease in heart failure. Malnutrition reflects a complex interplay of reduced protein reserves, immune dysfunction, anemia, and catabolic metabolism, all of which directly influence functional capacity, treatment tolerance, and recovery during acute decompensation. Unlike inflammatory markers, which may fluctuate rapidly in response to acute stress or infection, nutritional indices such as PNI and HALP integrate more stable physiological domains, including hepatic protein synthesis, hematopoiesis, and immune competence. As a result, nutritional status may serve as a more comprehensive surrogate of biological vulnerability in patients with acute heart failure (3, 4, 7).

This study's findings are consistent with and extend prior evidence highlighting the prognostic importance of nutritional status in heart failure. In a recent comprehensive review, El-Sheikh et al. emphasized that nutritional indices such as PNI and geriatric nutritional risk index (GNRI) provide meaningful prognostic information across the spectrum of acute and chronic heart failure, independent of traditional inflammatory markers. These observations support the concept that malnutrition reflects a broader chronic disease burden, encompassing metabolic, immunological, and functional impairment (3, 7, 20). In this context, our results complement existing literature by demonstrating that nutritional indices not only predict mortality and adverse outcomes but also have strong discriminatory value for hospitalization length in patients with acute decompensated heart failure and reduced ejection fraction.

Moreover, the length of hospital stay represents a composite outcome influenced not only by biological severity but also by health-system–related factors such as response to therapy, functional recovery, discharge planning, and availability of post-discharge support. Patients with poor nutritional status may experience delayed decongestion, impaired rehabilitation, and increased susceptibility to complications, all of which can prolong hospitalization. Therefore, the association between nutritional indices and length of stay likely reflects both intrinsic biological vulnerability and reduced resilience to the demands of acute heart failure management (3, 4, 13, 14).

Elevated SII and SIRI scores were linked to prolonged LOS, reflecting an increased inflammatory burden. Yang et al. reported SII as predictive of poor outcomes in coronary artery disease (9), while Zhang et al. observed diminished prognostic value of SII in multivariate models, consistent with our results (11). Li et al. further emphasized SIRI's prognostic value in cancer but variable performance in cardiovascular disease (10).

The lack of major differences in demographics (e.g., age, comorbidities, EF) between LOS groups suggests biomarkers may be more sensitive predictors than traditional risk factors (7, 8). The observed sex difference, with more males in the short LOS group, is noteworthy. Meyer et al. reported better short-term outcomes in male HF patients, possibly due to differences in remodeling or adherence (21). Our findings raise the possibility that sex-specific responses to nutritional and inflammatory status influence LOS.

All 15 deaths occurred in the prolonged LOS group, linking extended hospitalization with worse prognosis (Table 1). Although limited by small numbers, this mirrors Sze et al., who found malnutrition and congestion strongly associated with mortality in HF (3). Elevated urea, creatinine, CRP, and troponin in prolonged stays further support the association between metabolic stress and poor outcomes (11, 22).

Clinically, PNI's high AUC (0.850) and cut-off of 40.51 suggest its potential as a risk stratification tool in ADHF. This study's results are consistent with previous reports highlighting the prognostic role of nutritional indices in cardiovascular diseases. In particular, Yurdam and Demir et al. demonstrated that PNI is a reliable predictor of hospitalization in HFmrEF patients, which supports the relevance of our findings. Although the retrospective design limited the availability of certain clinical parameters, such as exercise capacity and NT-proBNP levels, the strong association between PNI and hospitalization risk suggests that nutritional assessment should be integrated into future prospective studies (23). Routine assessment of PNI and HALP at admission may guide nutritional interventions to improve outcomes (4). While inflammation correlated with prolonged LOS, its weaker predictive power suggests nutritional optimization should be prioritized. Future multicenter prospective studies should evaluate dynamic changes in these biomarkers and test interventions targeting both malnutrition and inflammation.

### Limitations

This study provides valuable insights into the prognostic roles of nutritional and inflammatory biomarkers in acute decompensated heart failure (ADHF) patients with left ventricular ejection fraction (LVEF) < 50%. However, several limitations should be considered when interpreting the findings:

- Retrospective Design and Single-Center Setting: The study's retrospective design limits establishing causality between nutritional (HALP, PNI) and inflammatory (SII, SIRI) biomarkers and outcomes like LOS and mortality. Single-center design limits the generalizability of the findings and precludes causal inference between nutritional and inflammatory biomarkers and clinical outcomes. In this study, dataset focused primarily on hospitalization and short-term clinical outcomes. It is critical to consider survival analysis in future prospective studies. Due to the retrospective nature of the dataset, an etiological classification (ischemic/non-ischemic) could not be made. Selection bias may exist as only patients with complete records were included.
- Dichotomisation of LOS: Length of hospital stay was dichotomized based on the cohort's average value. Although this approach was chosen to enhance statistical robustness and clinical interpretability, dichotomization may result in loss of information compared with analyses using LOS as a continuous variable.
- Low in-hospital causal mortality: Mortality analyses should be interpreted cautiously, as the number of in-hospital deaths was low and these analyses were exploratory in nature. The success of the cardiac team in the treatment and management of heart failure is undeniable, but it should be noted that these data are limited and refer to a specific time period.
- Lack of Longitudinal Biomarker Data: Key heart failure biomarkers such as NT-proBNP were not included due to missing data in a substantial proportion of patients, and biomarker measurements were limited to admission values. The absence of longitudinal biomarker trajectories restricts the assessment of dynamic changes during hospitalization. In patients with LVEF <50%, NT-pro-BNP is important for diagnostic confirmation and prognostication. While these values were available in a portion of the patient population, they were not included in the analysis due to missing data in a significant portion of the entire cohort. Biomarker measurements (HALP, PNI, SII, SIRI) were derived from admission blood samples. Longitudinal data could provide insights into their prognostic value during hospitalization and recovery.
- Unexplored Gender Disparity: The higher proportion of males in the short LOS group (65.9% vs. 52.3%, *p* = 0.036) was not fully explored. Sex-specific differences in disease severity, treatment response, and biomarker profiles could influence outcomes and warrant prospective studies.
- Potential Confounding Factors: Despite adjustment for major clinical and biochemical confounders, residual confounding cannot be excluded. Important unmeasured factors such as frailty, sarcopenia or body composition, severity of congestion, functional status, and discharge-related or health-system factors may have influenced hospitalization duration. Although multivariate logistic regression adjusted for key confounders (age, gender, rhythm, glucose, BUN, ALT, CRP, troponin, EF), unmeasured variables like socioeconomic status, medication adherence, and frailty could influence LOS and biomarker associations.
- Lack of the Etiological Classification of Heart Failure: Finally, the study population included a mixed cohort of patients with HFmrEF and HFrEF, and the etiological classification of heart failure was not available. This heterogeneity may have diluted associations and should be addressed in future studies.

Future studies should address these limitations through prospective, multicenter designs, larger sample sizes for mortality analyses, longitudinal biomarker monitoring, and exploration of sex-specific responses to enhance the generalizability and clinical applicability of the findings.

## Conclusions

In patients with ADHF and LVEF <50%, poor nutritional status, reflected by low HALP and PNI scores, and heightened inflammatory burden are associated with prolonged hospitalization. Among the evaluated biomarkers, nutritional indices—particularly PNI—demonstrated independent and robust predictive value for shorter length of stay, highlighting their potential role in early risk stratification.

These findings should be considered hypothesis-generating and warrant confirmation in prospective, multicenter studies. Future research should incorporate comprehensive assessments of frailty, body composition, and longitudinal biomarker trajectories to better elucidate the dynamic interplay between nutritional status, inflammation, and clinical outcomes in acute heart failure. Integrating nutritional evaluation into routine clinical practice may ultimately contribute to more personalized management strategies and improved patient outcomes.

## Data Availability

The raw data supporting the conclusions of this article will be made available by the authors, without undue reservation.
